# The diagnostic performance of patient symptoms in screening for COPD

**DOI:** 10.1186/s12931-018-0853-5

**Published:** 2018-08-03

**Authors:** Kate M. Johnson, Wan C. Tan, Jean Bourbeau, Don D. Sin, Mohsen Sadatsafavi, for the Canadian Cohort of Obstructive Lung Disease (CanCOLD) study and the Canadian Respiratory Research Network, Jean Bourbeau, Wan C. Tan, J. Mark FitzGerald, Don Sin, Darcy Marciniuk, Dennis E. O’Donnell, Paul Hernandez, Kenneth R. Chapman, Robert Cowie, Shawn Aaron, F. Maltais, Jonathon Samet, Milo Puhan, Qutayba Hamid, James C. Hogg, Jean Bourbeau, Carole Jabet, Palmina Mancino, Wan C. Tan, Don Sin, Sheena Tam, Jeremy Road, Joe Comeau, Adrian Png, Harvey Coxson, Jonathon Leipsic, Cameron Hague, Mohsen Sadatsafavi, Teresa To, Andrea Gershon, Wan C. Tan, Harvey Coxson, Jean Bourbeau, Pei Zhi Li, Zhi Song, Yvan Fortier, Andrea Benedetti, Dennis Jensen, Wan C. Tan, Christine Lo, Sarah Cheng, Cindy Fung, Nancy Haynes, Junior Chuang, Licong Li, Selva Bayat, Amanda Wong, Zoe Alavi, Catherine Peng, Bin Zhao, Nathalie Scott-Hsiung, Tasha Nadirshaw, Jean Bourbeau, Palmina Mancino, David Latreille, Jacinthe Baril, Laura Labonté, Kenneth Chapman, Patricia McClean, Nadeen Audisho, R. Cowie, B. Walter, Ann Cowie, Curtis Dumonceaux, Lisette Machado, Paul Hernandez, Scott Fulton, Kristen Osterling, Shawn Aaron, Kathy Vandemheen, Gay Pratt, Amanda Bergeron, Denis O’Donnell, Matthew McNeil, Kate Whelan, François Maltais, Cynthia Brouillard, Darcy Marciniuk, Ron Clemens, Janet Baran

**Affiliations:** 1 Respiratory Evaluation Sciences Program, Collaboration for Outcomes Research and Evaluation, Faculty of Pharmaceutical Sciences, University of British Columbia, Vancouver, Canada; 2 Centre for Heart Lung Innovation (the James Hogg Research Centre), St. Paul’s Hospital, Vancouver, Canada; 3 Respiratory Epidemiology and Clinical Research Unit, McGill University, Montreal, Canada; 4 Centre for Clinical Epidemiology and Evaluation, Vancouver Coastal Health Institute, Vancouver, Canada; 5 Institute for Heart and Lung Health, Department of Medicine, The University of British Columbia, Vancouver, Canada

**Keywords:** Screening test, Population, Respiratory symptoms, Chronic obstructive pulmonary disease

## Abstract

It is recommended that screening for COPD be restricted to symptomatic individuals, but supporting evidence is lacking. We determined the performance of wheeze, cough, phlegm, and dyspnea in discriminating COPD versus non-COPD in a population-based sample of 1332 adults. Area Under the Receiver Operating Curves (AUC) indicated that symptoms had modest performance whether assessed individually (AUCs 0.55–0.62), or in combination (AUC for number of symptoms as the predictor 0.64). AUC improved with the inclusion of multiple other factors (AUC 0.71). Restricting screening to symptomatic individuals is unlikely to substantially improve the yield of general population screening for undiagnosed COPD.

## Introduction

Chronic Obstructive Pulmonary Disease (COPD) is a common inflammatory lung condition that is characterized by symptoms of shortness of breath, cough, and sputum production [1]. Although COPD is under-diagnosed in the community [2], several major guidelines, including from the influential US Preventive Services Task Force, have recommended against the use of spirometry to screen for COPD in asymptomatic individuals in the general population because the number-needed-to-screen (NNS) to prevent adverse disease outcomes is prohibitively large [3, 4]. Some have advocated for case finding strategies to improve the diagnosis rate in the community in a more cost-effective manner, for example by targeting spirometry only among symptomatic individuals [5]. However, many patients with undiagnosed COPD have mild disease and may have few (if any) respiratory symptoms [6], and individuals without COPD can experience symptoms similar to those of COPD patients [7]. In addition, symptomatic COPD patients tend to be diagnosed earlier [6] and are therefore removed from the pool of cases that would be detected through a screening program. We determined the diagnostic performance of patient symptoms for screening in the general population to assess whether the yield of screening could be improved by restricting it to the symptomatic population.

## Methods

We used data from the Canadian Cohort of Obstructive Lung Disease (CanCOLD) Study. CanCOLD was a prospective cohort study of 1332 adults ≥40 years who were sampled from the general Canadian population with multi-level sampling to ensure representativeness. Participants were followed for a maximum of 3 years with visits at 18-month intervals [8]. They reported their demographic information, smoking status and history, comorbidities, and respiratory symptoms at each visit using validated questionnaires. Diagnostic spirometry was performed at each visit and persistent airflow limitation was defined as post-bronchodilator FEV_1_/FVC < lower limit of normal. Participants were deemed to have undiagnosed COPD if they had persistent airflow limitation but did not report previous physician-diagnosed COPD, emphysema, or chronic bronchitis. Subjects with a previous diagnosis of COPD were excluded. Information was collected on the frequency or severity of cough, phlegm, and wheeze using three questions for each symptom. The responses were coded as a variable ranging from 0 to 3 for each symptom. Breathlessness was measured using the Medical Research Council dyspnea scale. We also assessed the total number of symptoms experienced by each participant (0–4 range).

First, we determined the independent associations between individual symptoms and the presence of undiagnosed COPD (v. no COPD) using a logistic regression model with symptoms as separate independent variables and adjusting for participant demographics, comorbidities, smoking status, and pack-years. Second, we assessed the diagnostic performance of symptoms when used individually to distinguish patients with undiagnosed COPD from non-COPD subjects. We evaluated the sensitivity and specificity of each symptom at different thresholds (i.e., 0, 1, 2, or 3) for defining a patient as symptomatic. We fitted Receiver Operating Characteristic (ROC) curves to determine the Area Under the Curve (AUC) for each symptom individually, as well as their combined performance using the total number of symptoms. Finally, we used the AUC of the above-mentioned logistic regression model to assess the performance of all individual symptoms and covariates together. An AUC of 0.5 indicates the model has no discriminatory ability. Generalized estimating equations were used in all models to account for clustering of observations within individuals.

Ethics approval for CanCOLD was obtained from the relevant institutional review board at each study site. Written informed consent was obtained from all participants prior to study entry.

## Results

The mean age of the sample was 67.4 years [SD 9.7], 44% were females, and 40% of the participants had three study visits. The overall prevalence of undiagnosed COPD was 26%; 95% had mild to moderate disease based on the GOLD spirometric grading system [1]. The regression model indicated that reporting wheeze, dyspnea, and cough on most days were independently related to the presence of undiagnosed COPD (Table 1). However, symptoms alone had poor performance in identifying patients with undiagnosed COPD. Almost all symptoms, regardless of the severity, had sensitivities and positive predictive values less than 50% (Table 2).

**Table 1:** Association between symptoms and other patient characteristics with the odds of having undiagnosed COPD (v. no COPD)

	OR	95% CI	*p*-value
Cough			
1 (vs.0)	1.08	0.86–1.36	0.49
2 (vs.0)	1.38	0.93–2.04	0.11
3 (vs.0)	1.35	1.09–1.68	0.01
Wheeze			
1 (vs.0)	1.34	1.03–1.73	0.03
2 (vs.0)	1.74	1.38–2.20	< 0.01
3 (vs.0)	1.77	0.93–3.38	0.08
Phlegm			
1 (vs.0)	1.21	0.89–1.66	0.23
2 (vs.0)	1.35	0.77–2.36	0.30
3 (vs.0)	1.24	0.93–1.65	0.14
Dyspnea			
2 (vs.1)	1.26	1.05–1.50	0.01
3 (vs.1)	2.05	1.30–3.22	< 0.01
4 (vs.1)	1.52	0.76–3.03	0.24
5 (vs.1)	3.35	1.88–5.97	< 0.01
Age^a^	0.83	0.74-0.94	< 0.01
Female (vs. male)	1.03	0.81–1.30	0.82
BMI^a^	0.78	0.68-0.89	< 0.01
Caucasian (vs. non-Caucasian)	2.39	1.28–4.48	0.01
Comorbidities			
1 comorbidity (vs. 0)	0.93	0.76–1.14	0.51
2 comorbidities (vs. 0)	0.75	0.48–1.18	0.21
Smoking between visits (vs. no)	1.16	0.96–1.41	0.13
Smoking Pack-Years			
20–40 (vs. < 20)	2.09	1.53–2.86	< 0.01
> 40 (vs. < 20)	3.09	2.22–4.32	< 0.01

*BMI* body mass index, *CI* confidence interval, *OR* odds ratio

^a^Variables were converted to z-scores in the regression model

**Table 2:** Prevalence of each symptom severity category in the whole population (‘*Prev*’) across all study visits, and the prevalence of undiagnosed COPD (‘*COPD+*’) within that symptom severity category. Sensitivity (‘*TP*’), specificity (‘*TN*’), positive predictive value (‘*PPV*’), and negative predictive value (‘*NPV*’) of each symptom when used alone to classify undiagnosed COPD (v. no COPD) using different severity thresholds

Symptom severity	Cough^a^	Wheeze^b^	Phlegm^c^	Dyspnea^d^	Total Symptoms^e^
	*Prev, COPD+*	*Prev, COPD+*	*Prev, COPD+*	*Prev, COPD+*	*Prev, COPD+*
0	72, 23%	78, 21%	83, 24%	70, 23%	45, 17%
1	12, 29%	10, 42%	6, 30%	26, 33%	29, 28%
2	3, 35%	10, 49%	1, 47%	3, 56%	16, 33%
3	13, 42%	2, 51%	9, 39%	1, 30%	7, 50%
4				< 1, 67%	4, 52%
	*TP, TN* *PPV, NPV*	*TP, TN* *PPV, NPV*	*TP, TN* *PPV, NPV*	*TP, TN* *PPV, NPV*	*TP, TN* *PPV, NPV*
0 vs. > 0	37, 76% 36, 77%	39, 84% 46, 79%	24, 85% 37, 76%	40, 74% 35, 77%	71, 50% 34, 83%
≤1 vs. > 1	24, 88% 41, 76%	23, 91% 49, 77%	16, 91% 40, 75%	7, 98%, 52, 75%	41, 78% 40, 79%
≤2 vs. > 2	21, 90% 42, 76%	4, 99% 51, 74%	14, 92% 39, 75%	1, 99%, 38, 74%	21, 93% 51, 77%
≤3 vs. > 3				< 1, > 99% 67, 74%	7, 98% 52, 75%

*Prev* prevalence, *COPD+* undiagnosed COPD, *TP* true positive (sensitivity), *TN* true negative (specificity), *PPV* positive predictive value, *NPV* negative predictive value

Patients were asked, since your last visit:

^a^1) Do you usually cough when you don’t have a cold? 1a) Are there months you cough most days? 1b) Do you cough most days for as much as 3 months?

^b^2) Have you had any wheezing or whistling in your chest? 2a) Do you only have wheezing or whistling when you have a cold? 2b) Have you had an attack of wheezing or whistling that made you short of breath?

^c^3) Do you usually have phlegm in your chest when you don’t have a cold? 3a) Are there months you have phlegm most days? 3b) Do you hav e phlegm most days for as many as 3 months?

^d^Scores on the Medical Research Council (MRC) Dyspnea scale are subtracted by 1

^e^The sum of the number of individual symptoms that participants reported

If screening was applied to the general population (a “blind” screening approach), the NNS to detect one COPD case would be 3.8. If screening was restricted to individuals who reported symptoms, the NNS would be 3.0, but compared with the “blind” approach, an additional 17% of individuals with persistent airflow limitation would be missed.

**Fig. 1:**
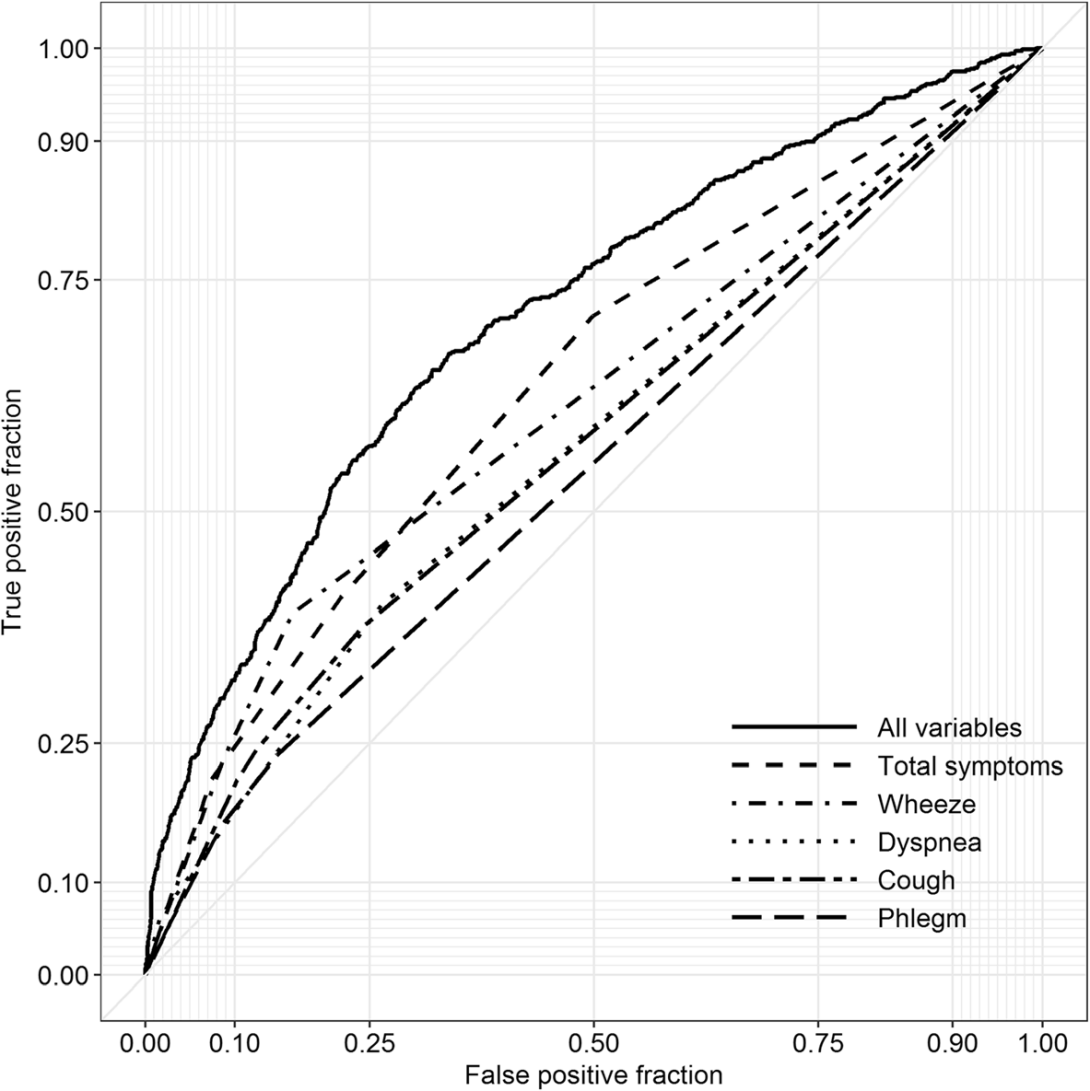
Receiver operating characteristic (ROC) curves for a model with all of the symptoms and covariates included (‘All variables’), as well as for each of the symptoms individually and the total number of symptoms reported by study participants (‘Total symptoms’)

The ROC curves indicated that wheeze had the best performance among all symptoms (AUC = 0.62), followed by cough and dyspnea (each AUC = 0.57), and phlegm (AUC = 0.55). The total number of symptoms performed marginally better than any symptom alone (AUC = 0.64). AUC improved by ~ 0.06 when each of the symptoms was combined with smoking history (measured as pack-years), resulting in an AUC of 0.67 for a model that included wheeze and pack-years. The model that included all individual symptoms and covariates improved the AUC to 0.71 (Fig. 1).

## Discussion

Our results indicate that symptoms are relatively poor at discriminating undiagnosed COPD patients from non-COPD subjects in a general population. The use of symptoms for screening is unlikely to significantly improve the diagnostic yield compared with “blind” screening in the general population. These data highlight the apparent paradox in finding strong associations between symptoms and undiagnosed COPD, yet poor diagnostic performance when symptoms are used to diagnose these “hidden” COPD cases. This observation is consistent with the well-established notion that a predictor can be strongly associated with an outcome while still being a poor classifier of that outcome [9]. Association models are useful for evaluating relationships at the population level, but classification models are more relevant to decisions at an individual level, specifically whether a test (e.g., the presence of a symptom) can detect the underlying disease state (e.g., undiagnosed COPD).

Previous studies have evaluated the merits of opportunistic case detection based on patient characteristics at the point of care [10, 11]. They generally found that patient characteristics and symptoms have modest capacity in detecting undiagnosed COPD [10–12]. A unique feature of our study is its population-based sample, which provides new evidence on whether the yield of population-based screening can be improved if symptoms are considered in the inclusion criterion (e.g., through advertisements for referral of symptomatic individuals for lung function testing). Although the costs and benefits of early intervention between symptomatic and asymptomatic patients were not considered here, our results do not support the use of symptoms in “case finding” for COPD. Symptoms should be used in conjunction with other characteristics such as pack-years of smoking to improve the diagnostic performance. Future studies should evaluate the cost-effectiveness of this approach considering the long-term outcomes associated with earlier diagnosis of COPD.

## Abbreviations

AUC: Area Under the Curve
CanCOLD: Canadian Cohort of Obstructive Lung Disease
COPD: Chronic Obstructive Pulmonary Disease
FEV_1_: Forced Expiratory Volume in 1 s
FVC: Forced Vital Capacity
GOLD: Global Initiative for chronic Obstructive Lung Disease
NNS: Number Needed to Screen
ROC: Receiver Operating Characteristic curves
SD: Standard Deviation
